# Antivenom availability, delays and use in Australia

**DOI:** 10.1016/j.toxcx.2022.100145

**Published:** 2022-12-08

**Authors:** Geoffrey K. Isbister

**Affiliations:** Clinical Toxicology Research Group, University of Newcastle, New South Wales, Australia

**Keywords:** Antivenom, Venom, Snakebite, Diagnosis, Envenoming

## Abstract

Antivenom is the main treatment for snake envenoming and there are ongoing concerns about availability in resource poor regions of the world. However, effective antivenom treatment for snake envenoming requires more than improved availability of safe and efficacious antivenoms. Most importantly, antivenom must be administered as early as possible, and within 2–6 h of the bite in Australia. At the same time, it is also important that antivenom not be given to all patients indiscriminately with a suspected snakebite, because of the risk of anaphylaxis. Delays in the administration of antivenom are a significant impediment to effective antivenom treatment and can be divided into pre-hospital and in-hospital delays. These range from delays due to remoteness of snakebite, to delays in diagnosis and administration of antivenom once in hospital. In Australia, antivenom is readily available in most hospitals, and a large portion of patients present to hospital within 2 h of the bite. However, there is on average a further delay of 2.5 h before antivenom is administered. Early diagnosis with accurate bedside tests and rapid clinical assessment of patients with snakebite are key to improving the effective use of antivenom.

## Highlights

• Antivenom must be administered as early as possible.
• Delays in the administration of antivenom can be pre-hospital or in-hospital.
• In Australia there are delays in antivenom administrations despite ready availability and early hospital arrival.
• Early diagnosis and rapid clinical assessment are essential to improve antivenom use.

## Graphical abstract

**Figure:**
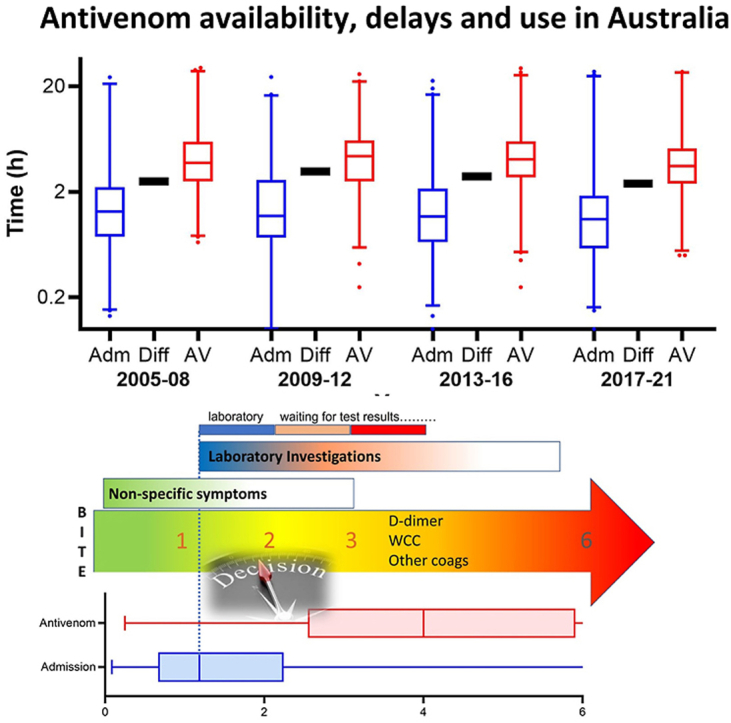

## 1 Introduction

Antivenom remains the key treatment for snake envenoming (Silva and Isbister, 2020), despite ongoing controversy about effectiveness (Isbister, 2010) and concerns about availability in resource poor regions of the world (Lalloo et al., 2002; Isbister and Silva, 2018; Potet et al., 2021). There has been significant focus on the lack of antivenoms available for snake envenoming in many parts of the world, most desperately in Africa, regions of Asia and the Americas (Lalloo et al., 2002). This ‘antivenom crisis’ has led to an international approach to improving access to antivenom and snakebite being recognised as a neglected tropical disease in 2017 (Williams et al., 2010; Gutierrez et al., 2010). The World Health Organisation and a number of groups have worked towards improving the quality of antivenoms and supporting manufacturers in producing more affordable and safe antivenoms. These initiatives will hopefully provide safe and effective antivenoms to most regions of the world, under-pinned by well-designed clinical trials.

Effective treatment of snake envenoming with antivenom requires more than improved availability of safe and efficacious antivenoms (Potet et al., 2021; Patikorn et al., 2022). Most importantly, antivenom needs to be administered to patients as early as possible, usually within hours of the bite. Numerous studies have demonstrated the increased effectiveness of early antivenom in preventing complications (Johnston et al., 2013, 2017a; Churchman et al., 2010; Trevett et al., 1995), and conversely delays in antivenom or treatment being associated with death (Magalhaes et al., 2022; Abdullahi et al., 2021; Margono et al., 2022). However, it is also important that antivenom not be given to all patients indiscriminately with a suspected snakebite. Even the safest antivenoms come with the risk of severe anaphylaxis (Isbister et al., 2008). The problem is that there will always be a delay in determining whether a patient is envenomed or not, and this needs to be balanced against the risk of antivenom administration to non-envenomed patients. The timely and judicious use of antivenom requires early and accurate diagnosis of systemic envenoming (Knudsen et al., 2021). Such decisions are based on early clinical features and/or available bedside or laboratory investigations (Knudsen et al., 2021; Wedasingha et al., 2020).

Delays in the administration of antivenom are a significant impediment to the effective use of antivenom (Silva et al., 2020; Cristino et al., 2021). There are numerous reasons for delays in antivenom administration, in addition to simply antivenom not being available in resource poor regions. Even if antivenom is available in the country or region, it may not be available in rural and remote locations, where snakebite is more common (Essafti et al., 2022). This requires a national or regional plan for the appropriate stocking of antivenom in locations where snakebite occurs.

Further sources of delay can be divided into pre-hospital and in-hospital delays, which will vary depending on the resources, culture and patient beliefs in different countries. The distance from the location of the snakebite to a health care centre, which stocks antivenom, is one of the most problematic delays. This is not confined to resource poor regions of the world (Johnston et al., 2017b), but is magnified in such regions due to poor transport and prehospital infrastructure (Silva et al., 2020; Cristino et al., 2021; Schurer et al., 2022). In many resource poor regions patients have often sought help from traditional medicine practitioners, rather than immediately attending hospital to receive antivenom (Margono et al., 2022; Schurer et al., 2022; Nann, 2021; Chaaithanya et al., 2021). Similarly, patients also use unsafe first aid methods in preference to being transported to hospital. Fortunately, this appears to be changing in some countries, with more people attending hospital for snakebite, rather than traditional healers (Silva et al., 2020; Wood et al., 2022). Finally, delays can occur because of patients' perceptions in regards to the severity of the bite, often leading to much longer delays (Cristino et al., 2021; Schurer et al., 2022). This is not confined to resource poor regions, and is a particular problem with snake handlers in many countries (Isbister et al., 2012).

In-hospital delays include both the time to make a diagnosis (assessment and investigation) and health system delays at all levels (Gutierrez et al., 2010; Silva et al., 2020). Delays between arrival to hospital and antivenom administration are a significant concern in hospitals that stock antivenom. Various studies of snakebite, including in countries with limited resources, show that a large proportion of snakebite victims arrive to hospital within hours of the bite (Silva et al., 2020; Johnston et al., 2017b; Patino et al., 2022). However, there is limited information on the time delay between admission and antivenom administration, most studies report either time to hospital or time to antivenom, but not both.

This paper will focus on delays in hospital for the administration of antivenom in Australia, because antivenom is readily available in most hospitals, and a large portion of patients present to hospital within 2 h of the bite.

## 2 Delays in antivenom administration

**Fig. 1:**
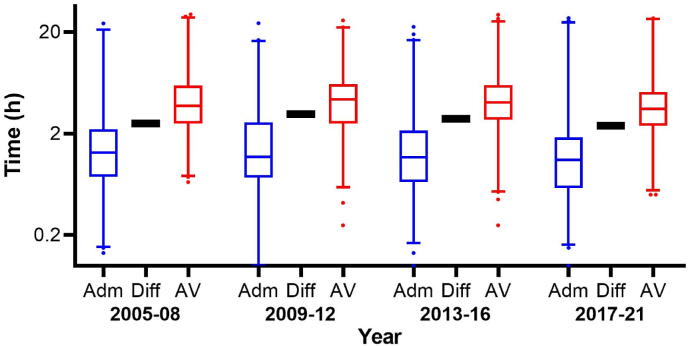
Box and whiskers plots of the time to hospital arrival, time to antivenom (AV) and the delay to antivenom from admission for 4 y periods between 2005 and 2021. Adm – time from bite to hospital admission; Diff – time difference between admission and antivenom administration; AV – time from bite to antivenom administration. Data taken from 1068 patients recruited to the Australian snakebite project (2005–2021), who were administered antivenom (Johnston et al., 2017b; Isbister et al., 2022).

A number of studies of Australian snake envenoming have demonstrated the effectiveness of early antivenom (<3 h) in preventing myotoxicity, neurotoxicity and acute kidney injury (AKI) (Johnston et al., 2013, 2017a; Churchman et al., 2010). Unfortunately, antivenom is rarely administered within this 3 h period, and this delay has not changed for at least 10 years in Australia (Johnston et al., 2017b). The median time to antivenom is 4 h, with a delay of 2.5 h on average between hospital arrival and antivenom administration (Fig. 1). In a recent study from the United States, the median time to antivenom was 3 h, but there was no details on the time to hospital arrival (Ruha et al., 2022). Despite, immediate availability of antivenom in many developed countries, a large proportion of patients still do not receive antivenom within 3 h.

It is well recognised that antivenom administration can result in early systemic hypersensitivity reactions. This is severe anaphylaxis in 3–5% of patients with Australian commercial antivenoms (Isbister et al., 2008), or much higher rates of reaction with antivenoms from other parts of the world (Silva et al., 2020; Giles et al., 2022). Therefore, a major issue in the treatment of snakebite is balancing the greater effectiveness of antivenom, when given early in patients with systemic envenoming, versus adverse effects occurring in patients in which antivenom is not indicated. Of 755 snakebite patients recruited to the Australian Snakebite Project given antivenom over 10 years, 49 were not envenomed, and of these 1 in 5 developed an allergic reaction unnecessarily (Johnston et al., 2017b). Studies in Sri Lanka demonstrate a similar problem with non-envenomed patients receiving antivenom and developing hypersensitivity reactions (Silva et al., 2020).

Concerns about non-envenomed patients receiving antivenom has led to a practice promoted for decades in Australian, in which antivenom is not given to patients until envenoming is definitely established (White, 1998; Isbister et al., 2013a). This has traditionally reinforced an approach in which, prior to antivenom being administered, there is thorough assessment and investigation first. Confirmatory laboratory investigations (e.g. coagulation studies) are regarded as essential, and determination of the snake species is important (i.e. snake venom detection kits) (Currie, 2004), prior to antivenom administration. Unfortunately, this approach has contributed to delays in antivenom being administered to patients. Delays due to waiting for results of diagnostic investigations, transfer to a second hospital for coagulation studies, or waiting for the development of clinical effects to determine the snake type. In many cases patients are transferred from one hospital, despite the availability of antivenom at the first hospital, to another hospital for laboratory investigations. This can delay treatment by 3–6 h.

On average there was a delay of 2.5 h between arriving in a health care facility in Australia and antivenom administration, and this has not changed over the last 17 years (Fig. 1). The median time to arrival in hospital was 1.2 h (Interquartile range [IQR]: 0.67–2.3 h), but the median time to antivenom was 4 h (IQR: 2.6–5.9 h). Half of the patients received antivenom >4 h post-bite and 24% got antivenom >6 h post-bite, many due to inter-hospital transfer.

Addressing these delays of 2–6 h before the administration of antivenom from the arrival to hospital is pivotal to increasing antivenom effectiveness in reducing myotoxicity, neurotoxicity and acute kidney injury. The focus needs to move to simply identifying patients who are envenomed and initiating antivenom treatment, rather than attempting to determine the type of snake and the clinical effects first.

Unique to Australia is the use of pressure bandaging with immobilisation in most snakebite patients, with the aim to delay the onset of systemic envenoming (Pearn et al., 1981). Pressure bandages are either applied by the patient, ambulance services or on arrival to hospital. However, one study found that the majority of patients already had evidence of envenoming on admission, despite the application of a pressure bandage (Ireland et al., 2010). Therefore, it cannot be assumed that the presence of a pressure bandage has prevented envenoming. Irrespective, current recommendations are for careful observation when the bandage is removed and repeat bloods performed 1 h after removal (TherapeuticGuidelines. Toxicology and Toxinology, 2020). There is no evidence that pressure bandages are harmful (Canale et al., 2009) or delay patients receiving definitive treatment with antivenom (Ireland et al., 2010).

## 3 Polyvalent versus monovalent antivenom

All antivenoms are polyclonal, being mixtures of antibodies raised in large mammals (e.g. horse, sheep), against multiple toxins in a snake venom. They are monovalent if raised against a single snake species and polyvalent if raised against multiple snakes species or groups of snakes, usually from a distinct geographical region (Silva and Isbister, 2020). The advantage of monovalent antivenoms are that they are specific for a group of snakes (i.e. species or genus) and therefore lower in volume with less protein, so potentially less risk of systemic hypersensitivity reactions. The disadvantage is that they will be ineffective or less effective if given for the wrong snake. Polyvalent antivenoms mitigate against this problem, but are larger in volume with increased risk of hypersensitivity reactions (Silva and Isbister, 2020).

There is a polyvalent and multiple monovalent snake antivenoms commercially available in Australia. In the past monovalent antivenoms were administered in preference to polyvalent antivenom, and the specific antivenom was guided by the results of a commercial snake venom detection kit (sVDK) (White, 1998; Coulter et al., 1980). This required a swab of the bite site for the test and further time taken to do the venom detection kit. A decision was then made as to which monovalent antivenom should be given based on the sVDK, geographical location and the clinical effects. This appeared to be an excellent approach, allowing a smaller and potentially safer dose of antivenom containing antibodies specific for the snake type. Outside of Australia, many perceived this to be a superior approach and various venom detection assay systems were subsequently developed in some countries (Liu et al., 2018; Long et al., 2021; Maduwage et al., 2020). Others recognised that there were issues with snake venom detection, particularly enzyme immunoassays, which do not always differentiate venoms of related snakes, because of non-specific reactions and cross-reactivity (Minton, 1987).

Despite decades of use of the sVDK in Australia, recent research found that the commercial sVDK was less reliable than initially reported, with a high false positive rate, and an incorrect or inconclusive result in one in six envenomed patients (Johnston et al., 2017b). The major issue is that the sVDK was incorrectly used in non-envenomed patients or there was operator error, rather than its correct use to determine the correct antivenom in envenomed patients (Nimorakiotakis and Winkel, 2016). In a study of over 1000 patients, the sVDK was inappropriately done in 364 non-envenomed patients and was falsely positive in 133 of the 364 patients (36%). Most concerningly, nine of these non-envenomed patients received antivenom based on the false positive result (Johnston et al., 2017b). Despite decades of education, health care staff continued to incorrectly assume that a positive result meant the patient was envenomed.

For envenomed patients, the sVDK was also problematic, based on results from the same study (Johnston et al., 2017b). For 597 envenomed patients in which the snake identity was confirmed by a separate venom specific enzyme immunoassay in blood or by expert identification, a bite site sVDK was positive for the incorrect snake in 5%, negative in 8% and inconclusive in 4%. In another study of confirmed tiger snake (*Notechis scutatus*) bites, the bite site sVDK was positive for brown snake (*Pseudonaja* spp.) in 5 of 44 cases, and in three of these brown snake antivenom was incorrectly administered (Isbister et al., 2013b). Fortunately for these patients administered the incorrect monovalent antivenom, it has now been recognised that Australian commercial antivenoms are all in fact polyvalent (O'Leary and Isbister, 2009). All terrestrial snake antivenoms are raised in a single horse population, immunised by the five major terrestrial snake genera, producing a polyvalent antivenom. The only difference between the monovalent antivenoms is their volume, because larger volumes are required for snakes that inject larger amounts of venom – taipan (*Oxyuranus* spp.), death adder (*Acanthophis* spp.) and mulga snake (*Pseudechis australis*). (O'Leary and Isbister, 2009; O'Leary et al., 2007).

The realisation in Australia that commercial antivenoms are polyvalent and that the sVDK was unreliable, has led to a new approach in the use of antivenom. Focus is now placed on diagnosing envenoming per se in patients, rather than attempting to identify the specific snake involved (TherapeuticGuidelines. Toxicology and Toxinology, 2020). Envenomed patients are then administered either two monovalent antivenoms (brown and tiger snake), which covers the majority of snakes in most parts of the country, or the much higher volume polyvalent antivenom, if there is a high likelihood of a taipan, death adder or mulga snake, based on geography or in snake handlers (Johnston et al., 2012, 2013, 2017a; Isbister et al., 2012). This has simplified the use of antivenom and reduces the risks of the incorrect antivenom being administered, or non-envenomed patients receiving antivenom. However, this puts more emphasis on developing diagnostic testing to identify systemically envenomed patients.

## 4 Addressing delays in antivenom administration

**Fig. 2:**
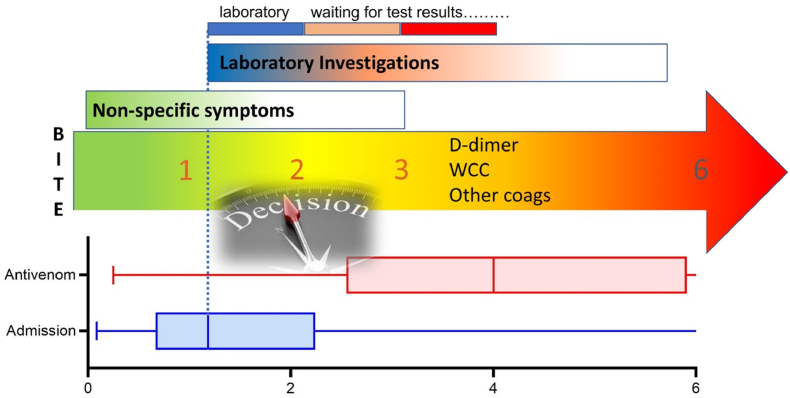
The golden 6 h of envenoming; WCC – white cell count. (For interpretation of the references to colour in this figure legend, the reader is referred to the Web version of this article.)

In Australia, the focus now needs to move toward early decision making about whether patients are envenomed, and therefore require antivenom. This can be thought of as the ‘golden’ first 6 h after a snakebite - a crucial time during which antivenom can prevent complications – most critically in the first 3 h (Fig. 2). In the past, this time has often been spent waiting on laboratory results and specific signs of envenoming to become evident. Such a decision may be difficult because the only evidence of envenoming may be non-specific systemic symptoms (nausea, vomiting, headache, diarrhoea and abdominal pain), and currently available routine bloods (D-dimer, white cell count and clotting studies). Even if these laboratory tests are turned around rapidly, they may not be diagnostic and take a minimum of an hour to be processed. This means that currently in Australia we rely on the clinical assessment on admission. In the future we need rapid point of care or bedside investigations for venom or other early biomarkers of envenoming to shorten this time (Isbister et al., 2020; Maduwage et al., 2014).

Early clinical assessment can be difficult, particularly for medical officers in rural and remote hospital settings, even in Australia. Although antivenom is available at these sites, there is limited pathology testing and often limited experience with snake envenoming. This can be addressed by early involvement of an expert in clinical toxicology via the Poison Centre, enabling early decisions regarding the administration of antivenom. This also avoids patients being transferred, creating long delays before antivenom administration. If patients have clear evidence of systemic envenoming with generalised symptoms, then antivenom can be administered at the primary site, then the patient transferred for further assessment and management.

Delays also occur in regional and larger urban hospitals, in which there are laboratory facilities. Most blood tests take at least 1 h to be analysed and reported, and this is usually only when the treating doctor chases and checks the blood results. The rapid analysis and reporting of blood tests for snakebite in Australia is not often streamlined, mainly because of the uncommonness of the presentation. In this case, far more importance should be placed on the initial clinical presentation of the patient, and if they have evidence of systemic envenoming, a decision to administer antivenom should be made prior to blood test results. Even an additional hour may significantly reduce the effectiveness of antivenom.

A further problem, specific to coagulation studies, is that blood collected from patients with venom induced consumption coagulopathy (VICC) does not clot i.e. prothrombin time/international normalised ratio is unrecordable. This means that laboratory technicians unfamiliar with snakebite (e.g. general and not haematology/coagulation laboratory technicians working after-hours) and modern automated coagulation analyses will delay results because they are unusually very abnormal. Early communication between the treating clinician and laboratory scientists is essential to prevent this occurring.

## 5 Early diagnostic testing

An important area for improvement in antivenom delivery is developing accurate bedside tests for systemic envenoming that can be used to make decisions almost immediately on arrival to hospital. A range of laboratory and bedside investigations have been used in the management of snake envenoming, but in general they have poor sensitivity and/or specificity, or are not available in most resource poor regions. The 20 min whole blood clotting test (WBCT-20) is the most widespread bedside test, despite minimal evaluation of its diagnostic accuracy, prior to its introduction (Isbister et al., 2013c). One study in Sri Lanka found that it has only moderately good sensitivity and specificity, even if staff are trained and standardised tubes are used (Ratnayake et al., 2017). More recently a study examining the best cut-off time for the WBCT, found that a 15 min WBCT was the most sensitive (Wedasingha et al., 2022).

**Fig. 3:**
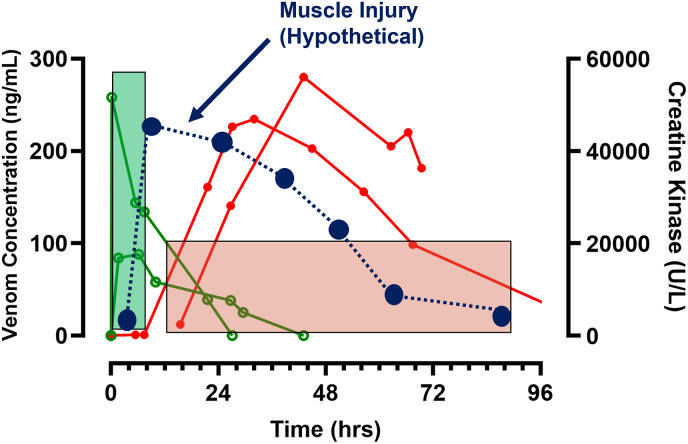
The pharmacokinetics and pharmacodynamics of venom concentrations (open green circles; ng/mL) and creatinine kinase (closed red circles; U/L) in two patients with severe myotoxicity. Hypothetical timing of muscle damage (closed blue circles and dashed line) shown. The time period in which antivenom is likely to be effective is < 6 h post-bite (filled green box) and ineffective >12 h post-bite (filled red box). (For interpretation of the references to colour in this figure legend, the reader is referred to the Web version of this article.)

In Australia, laboratory tests have been used routinely for the diagnosis of systemic envenoming in patients with suspected snakebites. The main problem with this approach is that many laboratory investigations are biomarkers of tissue injury or inflammation, and therefore only become abnormal after there is toxin-induced injury to specific organs (Ireland et al., 2010). For example, an elevated creatine kinase was recommended in the past as an indication for antivenom administration, to treat myotoxicity (White, 1998). However, numerous studies have demonstrated that there is a significant delay in increases of creatine kinase, and once this occurs it is well after the time in which antivenom is effective (Fig. 3). (Johnston et al., 2013; Johnston and Isbister, 2021) This makes theoretical sense because creatine kinase is released from cells after irreversible toxin-induced muscle injury, too late for antivenom to bind myotoxins and prevent such injury. For antivenom to be effective, it needs to bind to venom while it is still in the circulation (central compartment), before it diffuses to muscle tissue (Fig. 3). There are similar delays for other biomarkers, including the white cell count (Ireland et al., 2010) and several renal biomarkers (Ratnayake et al., 2019). Renal biomarkers are useful for the early identification of patients who develop acute kidney injury, for appropriate supportive care and renal replacement therapy, rather than antivenom administration.

A recent study investigated the diagnostic accuracy of a D-Dimer for VICC in Australian snakebite (Isbister et al., 2022). Although it found that an increased D-Dimer was a sensitive indicator of VICC, it was still only optimal between 2 and 6 h post-bite, making it difficult to administer antivenom within 3 h. Like other biomarkers, D-Dimer still measures an end-organ injury, in this case it indicates the activation of the clotting pathway, so a venom effect rather than the presence of venom.

The best diagnostic test for systemic envenoming is a test that measures or detects the presence of venom in blood. This is by definition synonymous with systemic envenoming. ELISA has been used to detect venom in patient serum (Kulawickrama et al., 2010), but currently available assays are confined to research laboratories, so not practical for clinical care of patients. A different approach is to quantify or detect individual toxins/toxin family groups in patient serum, which requires determining the most common toxin groups occurring in venoms across all snakes. There are four dominant protein families in snake venoms: phospholipase A_2_ (PLA_2_), snake venom metalloproteinases, snake venom serine proteases and three-finger toxins (Tasoulis and Isbister, 2017). It then requires developing a point of care test that can detect one or more of these dominant toxin families in patient serum.

PLA_2_ is the most common toxin family across snakes worldwide, including both vipers and elapids (Tasoulis and Isbister, 2017). Measurement of PLA_2_ activity has been used in two studies of snakebite patients to identify patients with systemic envenoming (Isbister et al., 2020; Maduwage et al., 2014). One small study of mainly Sri Lankan vipers demonstrated that increased PLA_2_ activity was diagnostic of Russell's viper envenoming, and to a lesser extent hump-nosed viper envenoming. More recently, a study of Australian snakebites showed that the PLA_2_ assay was diagnostic of envenoming, except for brown snake envenoming (Isbister et al., 2020). Further development of a bedside PLA_2_ test will be important, and potentially improve early diagnosis of systemic envenoming in snake bite.

## 6 Conclusion

There continues to be delays and inefficient use of antivenom, even when antivenom is available in hospital. Australia provides some useful insights into these issues, with an average of 2.5 h delay between hospital arrival and antivenom administration. Early diagnosis with accurate bedside tests and initial rapid clinical assessment of patients are essential to improving the effective use of antivenom. This is assisted by having access to expert advice and health care systems that expedite early decision making.

## Author credit

**Geoffrey Isbister:** Conceptualization, Methodology, Writing – original draft preparation and Editing.

## Ethical statement

This is a review article that does not require ethics.

## Declaration of competing interest

The authors declare that they have no known competing financial interests or personal relationships that could have appeared to influence the work reported in this paper.

## Data Availability

No data was used for the research described in the article.
